# Exploring risk signals of association between drugs and suicide: a retrospective investigation from 2004 to 2024

**DOI:** 10.3389/fpsyt.2026.1830964

**Published:** 2026-04-29

**Authors:** Zhilan Zhou, Junlin Diao, Jie Wan, Lurong Yu, Limei Liu

**Affiliations:** 1 Pharmacy Department, Chongqing Mental Health Center, Chongqing, China; 2 College of Traditional Chinese Medicine, Chongqing Medical University, Chongqing, China; 3 School of Chinese Materia Medica, Chongqing University of Chinese Medicine, Chongqing, China; 4 Department of Pharmacy, First People’s Hospital of Zigong City, Zigong, Sichuan, China

**Keywords:** FAERS database, intentional self-injury, neurotransmitter regulation, suicide risk, time to onset

## Abstract

**Background:**

Suicide is a serious public health issue associated with the interaction of biological, psychological and social factors. Although relevant studies are relatively mature, research on the association between drugs and suicide remains limited and lacks systematic analysis.

**Objective:**

This study aimed to explore the potential association signals between drugs and suicide-related adverse events (SAEs) using the U.S. Food and Drug Administration Adverse Event Reporting System (FAERS) database.

**Methods:**

This study collected data from the FAERS database spanning the first quarter of 2004 to the fourth quarter of 2024. We associated 10 suicide-related preferred terms with the primary suspected drug, employed a disproportionality method for risk signal detection, used cumulative distribution curves to assess time to onset characteristics after drug use, and conducted subgroup analyses by age and gender.

**Results:**

This study collected 247,657 reports of SAEs involving 193 drugs. The drug class most closely associated with SAEs were central nervous system medications; the majority of these drugs exhibited an early failure type, meaning that SAEs were more likely to occur during the initial stages of medication use. Among individuals <18 years old, the top three drugs by reported cases were montelukast, isotretinoin, and sertraline; hydrocodone/acetaminophen showed significantly higher ROR in individuals ≥65 years old. Quetiapine and paracetamol showed positive signals across all age groups, with ROR strength increasing with age.

**Conclusion:**

Significant differences exist in SAEs timing and associated drug classes across age groups and genders. These findings support targeted drug safety monitoring for high-risk populations. Combining multiple datasets in future research could deepen mechanistic understanding, improve risk assessment systems, and further ensure public drug use safety.

## 1 Introduction

Suicide is a serious worldwide public safety issue (1). According to the World Health Organization (WHO), more than 720,000 people die by suicide each year globally, and many more attempt suicide and intentional self-injury (2). Intentional self-injury is a strong predictor of suicide, especially among adolescents and young adults, with significant associations between the onset of early intentional self-injury and subsequent suicide attempt. A greater frequency of intentional self-injury is associated with a greater risk of suicide (3, 4). Some studies have shown that genetic factors play an important role in suicidal tendencies and individuals with a family history of suicide are at a higher risk of suicide (5). Psychological disorders such as depression, anxiety, and schizophrenia are closely linked to suicide (6), with the risk being significantly higher in patients with depression compared to the general population, especially during depressive episodes when suicidal ideation and behavior are common (6). Psychological distress is a key factor in suicide (7), as evidenced by a Kenyan study showing that individuals who have experienced psychological trauma are at a significantly increased risk of suicide, particularly if they have been subjected to emotional or physical abuse (8). A Danish study also suggests that emotional dysregulation may be associated with abnormal levels of neurotransmitters such as serotonin (5-HT) and dopamine (DA) in the brain, thereby further increasing the risk of suicide (9). Suicide is a complex outcome influenced by a combination of biological, psychological, social and other multidimensional factors.

Currently, there are numerous mature studies on the correlation between biological, psychological and social influences and suicide. However, the correlation between drugs and suicide is relatively limited and lacks systematic analysis. There is growing evidence that some drugs may be associated with suicide risk through direct or indirect mechanisms. Among them, psychotropic drugs such as antidepressants and antiepileptics, as well as cardiovascular and digestive drugs, may be linked to the occurrence of psychiatric symptoms such as depression and anxiety by affecting the release and regulation of neurotransmitters, thereby increasing the risk of suicide (10–14). Drugs such as glucocorticoids, immunosuppressants, and proton-pump inhibitors may also affect the central nervous system abnormalities in the patient during long-term use, which is linked to the occurrence of suicide-related adverse events (SAEs) (15–18). In addition, some studies have shown that the use of sleep-related medications is positively associated with suicidal ideation, planning, and attempts (19). However, in clinical practice, the associated risks linked to medications are often not given sufficient attention by clinicians.

Although there have been some studies on the association between drugs and suicide risk, most of these studies have limitations such as limited sample size and short study periods, which make it difficult to comprehensively and accurately reveal the complex relationship between drugs and suicide. The Food and Drug Administration (FDA) Adverse Event Reporting System (FAERS) database is an authoritative pharmacovigilance database containing a large number of adverse drug reaction reports (20). The database covers information on patients of different ages, genders, and races, providing a rich data resource for systematic research on the relationship between drugs and suicide risk. This study extracted data from the FAERS database from the first quarter of 2004 to the fourth quarter of 2024, aiming to comprehensively and systematically identify drugs associated with suicide risk and their risk characteristics through data mining and analysis. Through in-depth analysis of adverse reaction reports, we explore the temporal patterns, population differences, and potential mechanisms of action of drugs associated with suicide risk to provide a basis for the alert system. We hope to attract the attention of clinicians; and provide a reference for them to assess the risk of drug therapy and optimize prescribing decisions.

## 2 Method

### 2.1 Data sources and standardization

The data for this study was obtained from the FAERS database on the official website of the US FDA. The FAERS database is a public database accessible to all, and its data files contained seven datasets, namely patient patient demographics and administration (DEMO), drug details (DRUG), records of AEs (REAC), patient outcomes (OUTC), sources of reports (RPSR), start and end dates of therapy for the reported drugs (THER), and indications for drug usage (INDI). We downloaded the raw data from the first quarter of 2004 to the fourth quarter of 2024 and imported it into SAS 9.4 software for data cleaning, removing duplicate reports according to the official FDA recommendations. In this study, we coded the names of adverse events in the FAERS database using the Medical Dictionary for Regulatory Activities dictionary (MedDRA27.1) (21). Based on the Standard MedDRA Query (SMQ) classification system used for pharmacovigilance activities in the MedDRA medical dictionary, and by grouping the preferred terms (PTs) included in the analysis according to behavioral phase and severity, a total of 10 suicide-related preferred terms were identified.; subsequently, PT codes matching these PTs were extracted from the REAC, and the research dataset was constructed by linking this PT code dataset with the DEMO and DRUG tables. Meanwhile, and standardized the names of drugs in the database using the WHO drug dictionary (Sep 2024).

### 2.2 Study design

In this study, we identified the primary suspect drugs (PS) using the preferred terms (PT) screened based on the SMQ classification of suicide in MedDRA27.1: “assisted suicide, intentional self-injury, suicide attempt, suspected suicide attempt, suspected suicide, completed suicide, suicidal ideation, depression suicidal, suicidal behavior, self-injurious ideation.” The corresponding codes for these PTs are “10079105, 10022524, 10042464, 10081704, 10082458, 10010144, 10042458, 10012397, 10065604, 10051154”. The above PTs cover the entire spectrum from suicidal ideation to completed suicide, which may lead to heterogeneity of research results and needs to be considered in the interpretation of subsequent results. Additionally, we categorized each medication using used the World Health Organization’s Anatomical Therapeutic Chemistry (ATC) Classification (22) (https://atcddd.fhi.no/atc_ddd_index/).

### 2.3 Statistical analysis

We used four methods to analyze potential signals between drugs and SAEs: Reporting Odds Ratio (ROR), Proportional Reporting Ratio (PRR), Bayesian Approach (Information Component, IC), and Empirical Bayesian Geometric Mean (EBGM). ROR and PRR are widely recognized and easy-to-interpret proportional imbalance analyses, while IC and EBGM can be adjusted according to the reporting rate to detect potential signals more accurately (23). In this study, the four methods mentioned above were used in combination for signal detection. The selection of signal detection thresholds is based on the consensus of international pharmacovigilance research and the actual characteristics of FAERS data: a signal was considered present when the number of reports a ≥ 3, the lower limit of ROR 95% CI > 1, PRR ≥ 2, *χ^2^* ≥ 4, IC-2SD > 0, and EBGM05 > 2 (24–26). The formulas and specific criteria for the four methods are shown in the **Supplementary Table 1**.

Time to onset (TTO) of adverse events was defined as the time period between the date of occurrence of SAEs and the date of initiation of drug use. We assessed TTO using the median, interquartile range (IQR), and Weibull shape parameter (WSP). When *β* < 1 and the 95% CI < 1, SAEs frequency decreases over time, indicating an early failure type, meaning that events are more likely to occur early in the exposure period (early in drug administration). When *β* ≈ 1 and the 95% CI includes 1, SAEs frequency remains essentially unchanged, indicating a randomized failure type, meaning that event occurrence is not significantly associated with time and is randomly distributed. When *β* > 1 and the 95% CI > 1, SAEs frequency gradually increases, indicating a wear-off failure type, meaning that events are more likely to occur after long-term exposure (long-term drug accumulation) (27). Additionally, we assessed TTO by age and sex, with SAEs frequency gradually increasing, indicating wear-and-tear failure (27). We stratified the data by age and sex and used cumulative distribution curves to assess TTO characteristics after drug use.

## 3 Results

### 3.1 Basic characteristics

**Figure 1:**
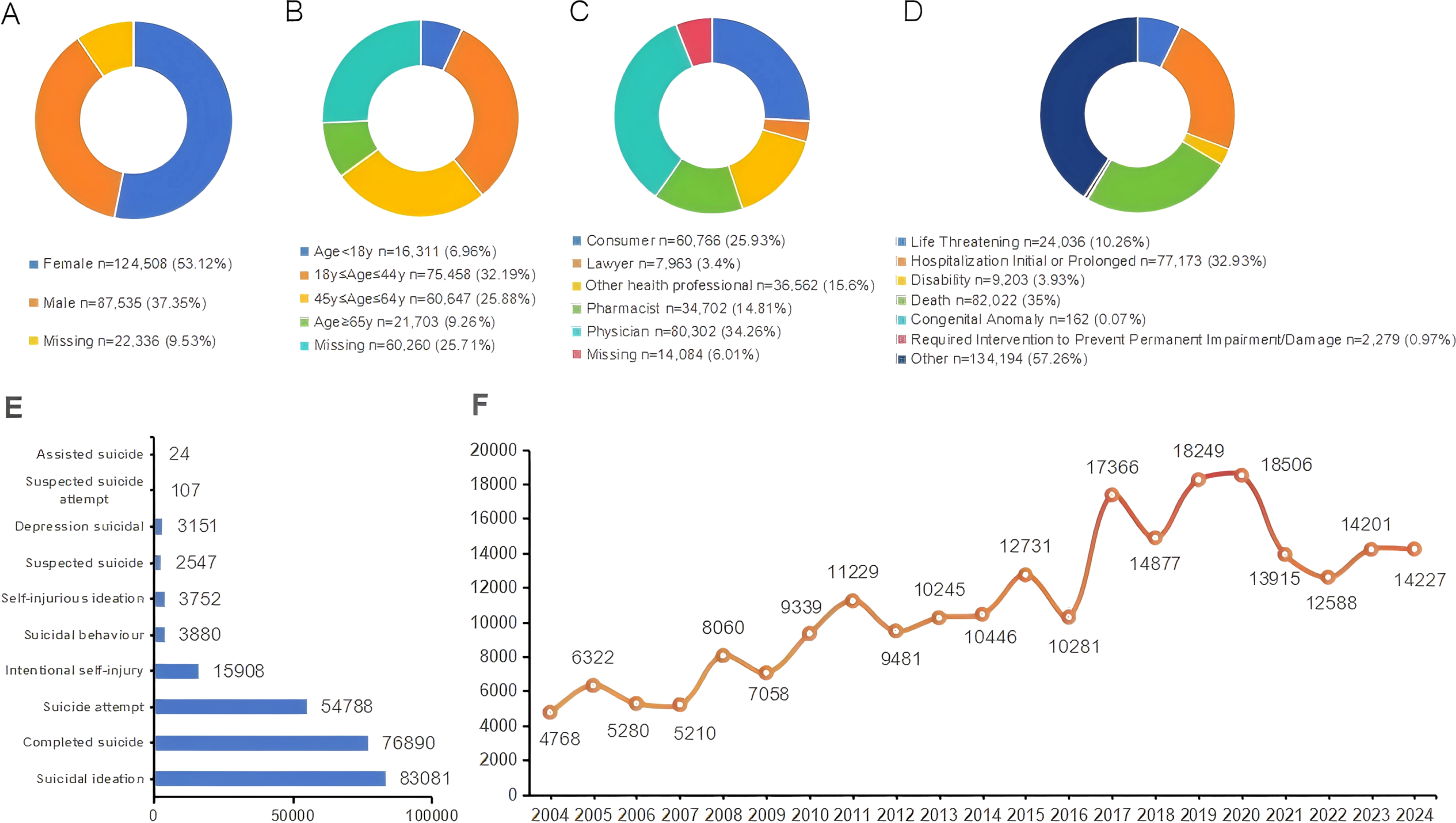
Basic information and patient characteristics of reports associated with suicide. **(A)** Distribution of patients by gender **(B)** Distribution of patients by age **(C)** Distribution of reporters **(D)** Distribution of patient outcomes **(E)** Distribution of PT associated with suicide or self-harm **(F)** Distribution of reporting years.

A total of 1,628 drugs were identified as “primary suspected drugs” associated with “suicide”, and 193 drugs were included in the analysis after signal detection. A total of 247,657 reports with SAEs were involved. **Figure 1** demonstrates the basic characteristics of the population included in the study, with a significantly higher proportion of female patients (53.12%, *n* = 124,508) than male patients (37.35%, *n* = 87,535). In aspect of age stratification, the highest number of patients were in the age group of 18–44 years (*n* = 75,458, 32.19%), followed by patients in the age group of 45–64 years (*n* = 60,470, 25.88%). In terms of reporting sources, over 60% of reports were from health professionals. Regarding serious outcomes, there were 82,022 (35.00%) deaths, 77,173 (32.93%) hospitalizations, 24,036 (10.26%) life-threatening patients, and 9,203 (3.93%) disabilities. The overall fluctuating upward trend in the number of SAEs reported is evident from the trend in the year of reporting.

### 3.2 Time to onset

**Figure 2:**
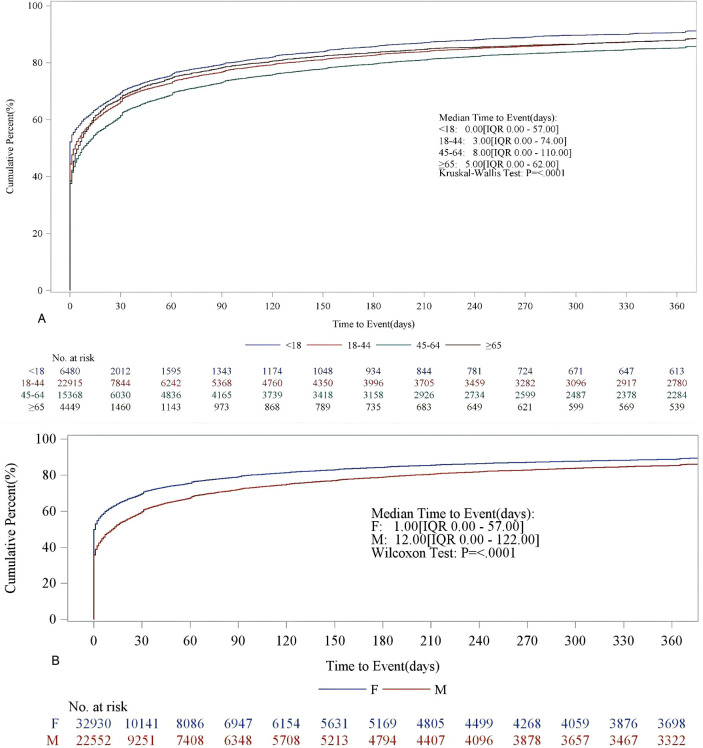
Cumulative incidence of SAEs occurrence (K-M curves). **(A)** Cumulative incidence of SAEs by age group (K-M curve) plot, **(B)** Cumulative incidence of SAEs by gender group (K-M curve) plot.

To ensure the accuracy of the analysis, we excluded all incorrect or missing data, resulting in fewer cases being used for further analysis than actual cases. **Table 1** lists the time of occurrence of SAEs for the top 30 drugs in terms of the number of reported cases. Sixteen drugs had a median time of occurrence of 0 days, while the top five with the highest median time of occurrence were montelukast (87.00, IQR: 5.00, 648.50), paroxetine (78.00, IQR: 3.00, 532.00), isotretinoin (76.00, IQR: 28.00, 228.00), gabapentin (51.00, IQR: 0.00,347.00), and esketamine (43.50, IQR: 8.00, 163.00). In terms of the distribution parameters, the top three drugs with the highest values of the scale parameter (*α*) were clonazepam (*α* = 577.93), montelukast (*α* = 382.49), and paroxetine (*α* = 377.80). Conversely, the top three drugs with the smallest values were paracetamol (*α* = 10.19), promethazine (*α* = 11.26), and ibuprofen (*α* = 15.07). All drug shape parameters *β* < 1 and 95% CI < 1, failure type is early failure, indicating that SAEs are more likely to occur during the initial exposure period (the early stages of drug administration). **Figure 2** presents the cumulative distribution curves of SAE incidence rates across different age and gender groups. Specifically, part A shows the cumulative incidence rates of SAEs across different age groups; there was a significant difference in the median time of occurrence in each age group (Kruskal-Wallis Test: *P* ≤ 0.0001). Among them, the median time to the occurrence of SAEs was shortest in minors, and the median time to the occurrence was longest in people aged 45–64 years. Part B shows the cumulative incidence of SAEs across different gender groups. There was a significant difference in the median time to SAE occurrence between the median occurrence time of males and females (Wilcoxon Test: *P* ≤ 0.0001). In particular, the median time to occurrence was longer in males (12 days) than in females (1 day).

**Table 1:** TTO and Weibull distribution parameters of SAEs for the top 30 drugs with reported cases.

Time to onset			Weibull distribution				Failure type
Drug Name	Cases	TTO (days)	Scale parameter		Shape parameter		
n	Median(IQR)	α	95% CI	β	95% CI	β	
Varenicline	3841	30.00(3.00,84.00)	84.40	80.19-88.84	0.73	0.71-0.75	Early failure
Quetiapine	2414	0.00(0.00,18.00)	268.30	233.88-307.78	0.54	0.51-0.57	Early failure
Sertraline	2194	0.00(0.00,18.00)	71.00	61.58-81.87	0.45	0.43-0.47	Early failure
Paroxetine	1993	78.00(3.00,532.00)	377.80	344.57-414.25	0.56	0.54-0.59	Early failure
Venlafaxine	1426	1.00(0.00,72.00)	259.21	221.41-303.46	0.49	0.46-0.52	Early failure
Promethazine	1383	0.00(0.00,0.00)	11.26	3.79-33.49	0.45	0.33-0.62	Early failure
Montelukast	1308	87.00(5.00,648.50)	382.49	340.23-429.99	0.54	0.51-0.56	Early failure
Alprazolam	1207	0.00(0.00,0.00)	117.79	77.35-179.37	0.35	0.31-0.38	Early failure
Escitalopram	1159	1.00(0.00,31.00)	84.42	71.31-99.95	0.50	0.47-0.53	Early failure
Olanzapine	1149	0.00(0.00,27.00)	192.77	159.62-232.81	0.53	0.50-0.57	Early failure
Isotretinoin	1141	76.00(28.00,228.00)	245.69	219.46-275.06	0.57	0.54-0.59	Early failure
Duloxetine	1128	31.00(2.00,193.50)	179.08	158.30-202.59	0.57	0.54-0.60	Early failure
Gabapentin	1122	51.00(0.00,347.00)	314.24	278.83-354.15	0.61	0.58-0.64	Early failure
Paracetamol	1111	0.00(0.00,0.00)	10.19	5.86-17.73	0.34	0.31-0.38	Early failure
Lorazepam	972	0.00(0.00,0.00)	77.84	48.53-124.83	0.38	0.34-0.43	Early failure
Citalopram	954	0.00(0.00,20.00)	83.58	66.84-104.51	0.44	0.41-0.47	Early failure
Fluoxetine	940	4.00(0.00,42.00)	115.69	94.84-141.12	0.45	0.43-0.48	Early failure
Mirtazapine	915	0.00(0.00,14.00)	46.19	37.95-56.20	0.52	0.48-0.55	Early failure
Ibuprofen	793	0.00(0.00,0.00)	15.07	7.52-30.23	0.47	0.38-0.59	Early failure
Aripiprazole	744	9.00(0.00,67.00)	131.63	109.67-157.98	0.54	0.51-0.58	Early failure
Zolpidem	734	0.00(0.00,1.00)	110.66	80.73-151.69	0.46	0.42-0.51	Early failure
Bupropion	732	5.00(0.00,33.00)	59.04	49.27-70.75	0.54	0.51-0.58	Early failure
Risperidone	687	0.00(0.00,25.00)	220.01	171.94-281.51	0.54	0.49-0.59	Early failure
Lamotrigine	648	0.00(0.00,22.50)	92.84	72.26-119.28	0.49	0.45-0.54	Early failure
Esketamine	588	43.50(8.00,163.00)	135.42	117.54-156.03	0.65	0.61-0.69	Early failure
Lisdexamfetamine	563	0.00(0.00,0.00)	94.24	67.64-131.29	0.59	0.51-0.68	Early failure
Clonazepam	550	0.00(0.00,21.00)	577.93	412.64-809.43	0.45	0.40-0.51	Early failure
Atomoxetine	461	31.00(4.00,147.00)	122.95	103.84-145.58	0.63	0.59-0.68	Early failure
Finasteride	405	39.00(7.00,318.00)	249.41	203.05-306.36	0.55	0.50-0.59	Early failure
Diazepam	387	0.00(0.00,0.00)	160.12	69.12-370.92	0.35	0.29-0.43	Early failure

### 3.3 Suicide-related risk signals

#### 3.3.1 Drug classes

In our study, the first-level categorization of suspected drug case reports according to ATC classification criteria revealed significant differences in the distribution of SAEs among different drug systems. As shown in **Supplementary Figure 1**, the most common drugs with reported SAEs belonged to the nervous system (ATC N, *n* = 175,686, 52.17%), followed by genito urinary system and sex hormones (ATC G, *n* = 24,876, 7.39%). **Supplementary Figure 2** demonstrates the distribution of the top 30 drug SAEs ranked by the number of cases. The results shows that SAEs such as completed suicide, suicide ideation, suicide attempt, and intentional self-injury can be associated with many drugs. Regarding suicide attempt, the top three most reported cases were quetiapine (*n* = 2682), varenicline (*n* = 2287), and paracetamol (*n* = 2400). Regarding suicidal ideation, the top four ranked cases were varenicline (*n* = 4497), duloxetine (*n* = 4226), paroxetine (*n* = 2654), and isotretinoin (*n* = 2337). For intentional self-injury, sertraline (*n* = 1061) and quetiapine (*n* = 1005) were the top two reported. In the top three rankings for the number of cases of completed suicide, paracetamol (*n* = 3955), hydrocodone/paracetamol (*n* = 3578), and amlodipine (*n* = 3153) were reported.

#### 3.3.2 Different age groups

**Figure 3:**
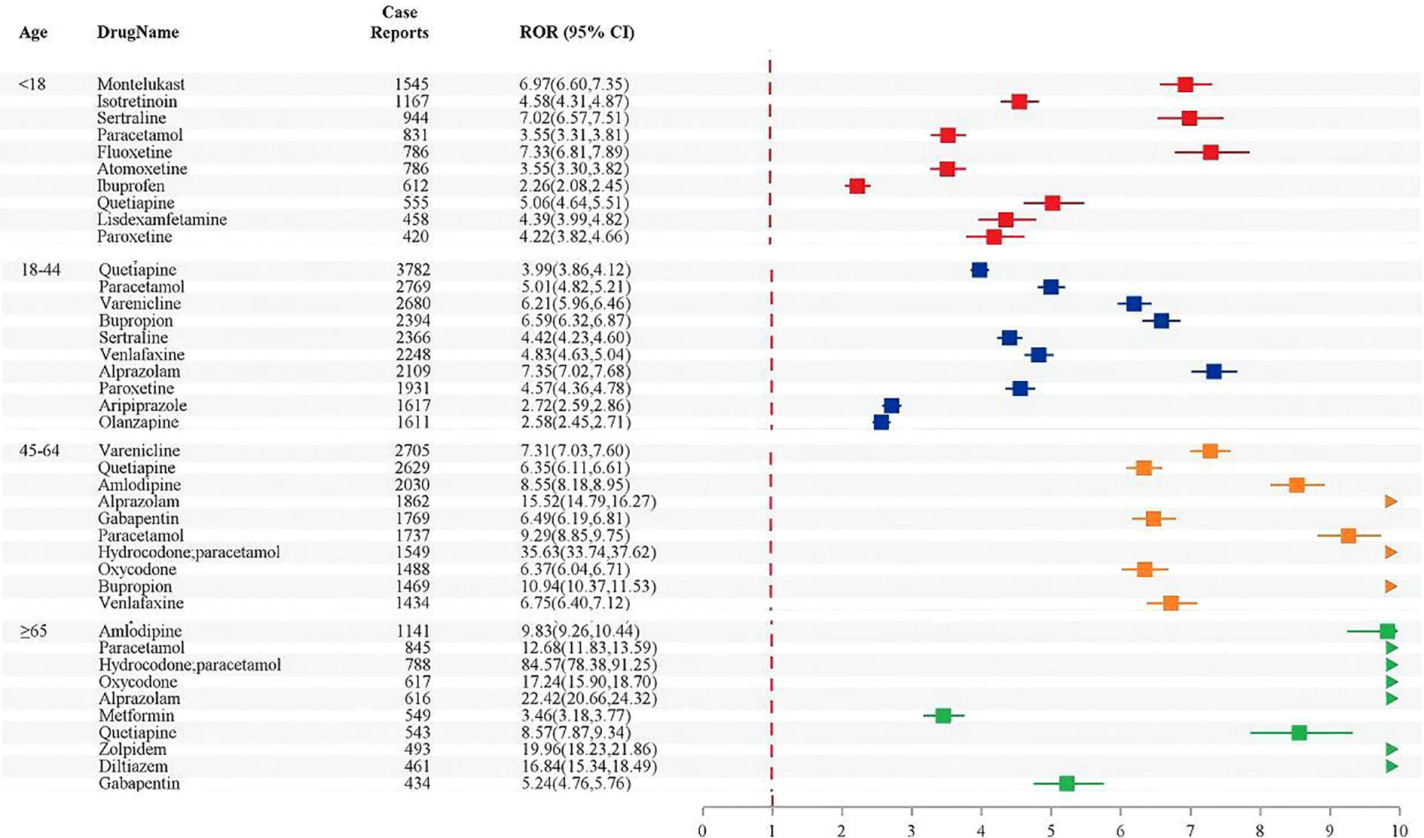
Top 10 drugs by number of reported SAEs by age group.

**Figure 3** presents the distribution characteristics of the top 10 drugs with the highest number of reported cases of SAEs in different age groups. In the <18 years old group, 8 drugs were nervous system drugs, 1 respiratory system drug, and 1 dermatological drug; the top three reported cases were montelukast (*n* = 1545), isotretinoin (*n* = 1167), and sertraline (*n* = 944). For 18–44 year olds, all drugs were related to the nervous system; the top three reported cases were quetiapine (*n* = 3782), paracetamol (*n* = 2769), and varenicline (*n* = 2680). Among those aged 45–64 years nine were nervous system drugs and one was a cardiovascular system drug, with the highest number of reported cases being varenicline (*n* = 2705), followed by quetiapine (*n* = 2629), and amlodipine (*n* = 2030). For those ≥65 years of age, there were eight nervous system drugs, one cardiovascular system drug, and one alimentary tract and metabolism drug; the top three reported cases were amlodipine (*n* = 1,141), paracetamol (*n* = 845), and hydrocodone/paracetamol (*n* = 788). Meanwhile, paracetamol and quetiapine had SAEs in all age groups and their ROR increased progressively with age; alprazolam’s ROR value in adults increased with age, reaching 22.42 in those ≥65 years old. Hydrocodone/paracetamol had an ROR value of 35.63 in 45–64 year olds, which was further elevated in those ≥65 years old to 84.29.

## 4 Discussion

The influencing factors of suicide have long been the focus of worldwide attention, and the drug factor is one of its influencing factors (28). Although some studies have confirmed the potential association between certain drugs and individual suicidal tendencies (29, 30), the studies are relatively limited and there is still a lack of comprehensive analysis of the complex relationship between drugs and suicide. In this study, we extracted data from the FAERS database on medications that have reported SAEs in the past 20 years and analyzed them at multiple levels to provide clinicians with a reference for assessing the risk of drug therapy and optimizing prescribing decisions.

In our study, the overall trend in the number of reports of SAEs showed a fluctuating upward trend over time, with significant gender and age differences observed in terms of population distribution and timing of occurrence. With regard to gender, the proportion of female patients (53.12%) was significantly higher than that of male patients (37.35%); the median time to onset for males (12 days) was longer than that for females (1 day); these results may indicate that women face a higher risk of experiencing SAEs following medication use. A South Korean cohort study found that women are at higher risk of suicide attempt, further corroborating our findings (4). Existing research suggests that female hormone levels fluctuate cyclically, and that changes associated with the menstrual cycle are linked to suicidal behavior (31–33). Furthermore, women are at higher risk of developing mood disorders, anxiety disorders, and depression, with depression being a major psychiatric risk factor for suicide (34, 35). The percentage of medications reported for the treatment of psychiatric disorders regarding SAEs in the present study was more than 50%, suggesting that there may be a need to focus on monitoring the use of medications in female patients with depression.

In addition, we found differences in TTO between different drugs. For example, montelukast (87.00, IQR: 5.00, 648.50), paroxetine (78.00, IQR: 3.00, 532.00), isotretinoin (76.00, IQR: 28.00, 228.00). These medications had high median TTO values, but with an extremely wide IQR suggesting that the time to occurrence of SAEs in the study population was more dispersed and that individual differences in medication use may be large. However, the median TTO of quetiapine, sertraline, and citalopram was 0 and the IQR was relatively short, suggesting that the patients most likely experienced SAEs on the same day as the drug was administered. This suggesting that clinical drug use requires enhanced monitoring in the initial stages. In addition, This study cannot rule out bias resulting from characteristics of the FAERS reporting system (such as data entry practices), which may be associated with recorded TTO values of 0 days.

In this study, nervous system drugs (ATC N) accounted for the highest percentage (52.17%). It is worth noting that these medications are commonly used in patients with mental disorders such as depression and schizophrenia, and individuals receiving these treatments are often associated with an elevated baseline risk of suicide. This study cannot rule out the potential influence of patient-related factors. For this population, underlying mental health conditions are closely associated with the occurrence of SAEs. In addition to nervous system drugs, cardiovascular system drugs, and digestive system drugs have also been found to be potentially associated with suicide risk (13), which is consistent with our findings. A recent real-world forensic study on drug-related completed suicide in Türkiye found that metabolic and cardiovascular drugs and antidepressants were the most commonly used drugs in suicide attempt, which is consistent with the results of this study (36). Notably, SAEs are particularly prominent with antidepressants, which is consistent with the 2004 FDA black box warning on antidepressants, stating that antidepressants may be associated with an increased risk of suicidal thoughts and behaviors in children, adolescents, and young adults (37). These findings suggest that when prescribing psychotropic medications and neuroactive non-psychotropic medications, clinicians should closely monitor patients for early signs of psychological abnormalities.

The types of medications that detected a positive signal varied across age groups. Among those under 18 years of age, the highest number of reports was on montelukast (*n* = 1545), followed by isotretinoin (*n* = 1167). In 2008, the FDA warned that montelukast may be associated with mood changes, suicidal thoughts, and suicidal tendencies (38). In 2020, they added a risk of neuropsychiatric events with a black box warning and restricted its use in allergic rhinitis (39). These warnings further support the results of the present study. Isotretinoin is FDA-approved for the treatment of acne but has black box warnings related to the risk of depression, suicide, and psychosis (40). This suggests that greater clinical attention needs to be paid to neuropsychiatric symptoms in minors using montelukast and isotretinoin. The top 3 drugs with the highest number of reported cases among those aged 18–44 years were quetiapine (*n* = 3782), paracetamol (*n* = 2769), and varenicline (*n* = 2680). Notably, paracetamol and quetiapine detected positive association signals in all age groups. However, a systematic review indicated that quetiapine is associated with a reduced risk of suicide (41); another clinical study demonstrated that treatment with cognitive behavioral therapy combined with quetiapine is associated with a reduced risk of suicide (42). Given the limitations of this study, including the failure to adjust for confounding factors such as mental disorder diagnoses, disease severity, and history of suicidal behavior, the high number of quetiapine reports among individuals aged 18–44 may be related to the large population of patients with mental disorders in this age group, the widespread clinical use of quetiapine, and the higher baseline risk of suicide among these patients. Theoretically, the association between acetaminophen and SAEs may be associated with its pharmacological characteristics, as the drug inhibits hepatic tryptophan 2,3-dioxygenase activity, an enzyme whose activity is associated with serotonin and 5-hydroxyindoleacetic acid (5-HIAA) levels in the brain (43, 44). Furthermore, studies have indicated that lower 5-HIAA concentrations are associated with more pronounced suicidal ideation, intentional self-injury, and suicide attempt (45). Additionally, a cohort study showed an association between acetaminophen overdose and intentional self-injury in individuals aged 60 and older (46). This is generally consistent with the present findings. The strength of association between hydrocodone/paracetamol (ROR = 84.57), alprazolam (ROR = 22.42) and SAEs was relatively high in those over 65 years of age. Elderly patients taking hydrocodone/paracetamol and alprazolam often have multiple underlying diseases and poor physical condition, which is the high-risk population of suicide, and the high ROR value may be related to the baseline risk of the population. Hydrocodone is an opioid-containing drug that is a central depressant and highly addictive (47). Opioids are often associated with suicides and accidental deaths, and suicides from intentional drug overdoses often involve prescription opioids (48). Alprazolam is a short-acting benzodiazepine (49). Studies have shown that its use is associated with an increased risk of suicide attempt (50),which supports the results of our study. In addition, there was a significant difference in the median time to SAEs in different age groups, which may be due to the different types of drugs used in various age groups.

There are some limitations to this study. First of all, all findings are preliminary associations derived from the FAERS spontaneous reporting database and cannot be used to infer a causal relationship between the drugs and SAEs. These associations require further validation through rigorously designed controlled epidemiological studies. Furthermore, this study did not adjust for confounding factors such as psychiatric diagnoses, disease severity, and prior suicidal behavior, which are associated with the misattribution of underlying disease risks to drug effects. This is one of the core limitations of this study. Second, the FAERS system is a spontaneous reporting mechanism and has inherent methodological flaws, including reporting bias, underreporting, lack of denominator data, coding variability, and the absence of clinical verification. These flaws are associated with data reliability and may be related to the misinterpretation of the observed signals. Finally, this study screened 10 suicide-related PTs based on the SMQ classification of MedDRA, covering the full spectrum from suicidal ideation and suicide attempt to completed suicide. However, the MedDRA terminology system exhibits hierarchical heterogeneity and multi-axial classification characteristics, which may be associated with classification bias for some SAE reports, introducing potential bias into the study. Despite these limitations, given the FAERS database’s extensive coverage and its advantages of international applicability and reproducibility, analyses of the association between drugs and SAEs based on this database system still hold significant clinical reference value.

## 5 Conclusion

This study systematically demonstrates the distributional characteristics of suspected drugs associated with suicide. There were significant differences between age groups and gender regarding the timing of occurrence of SAEs and drug classes. Quetiapine and paracetamol were identified as positive signals in all age groups. There is a need to focus on the use of montelukast and isotretinoin drugs in the underage population, while in the middle-aged and elderly population, the risk of hydrocodone/paracetamol use is higher and needs to be monitored. All findings in this study represent preliminary associations derived from the FAERS spontaneous reporting database; they do not establish a causal relationship between the drugs and SAEs; Future studies could incorporate rigorously designed controlled epidemiological studies, such as cohort studies and case-control studies, as well as real-world data, to further validate the validity of these association signals. Additionally, basic experimental research could be integrated to explore the potential biological pathways underlying the association between medications and SAEs, thereby refining the drug suicide risk assessment system.

## Data Availability

Publicly available datasets were analyzed in this study. This data can be found here: https://fis.fda.gov/extensions/FPD-QDE-FAERS/FPD-QDE-FAERS.html.
