# Supplementary material for: Exploring risk signals of association between drugs and suicide: a retrospective investigation from 2004 to 2024
Source: Front Psychiatry. 2026 Apr 29;17:1830964. doi: 10.3389/fpsyt.2026.1830964 (PMC13167444; doi:10.3389/fpsyt.2026.1830964)
Supplement: Supplementary file 1 [file Supplementaryfile1.docx]

Supplementary Material

# Supplementary Figures


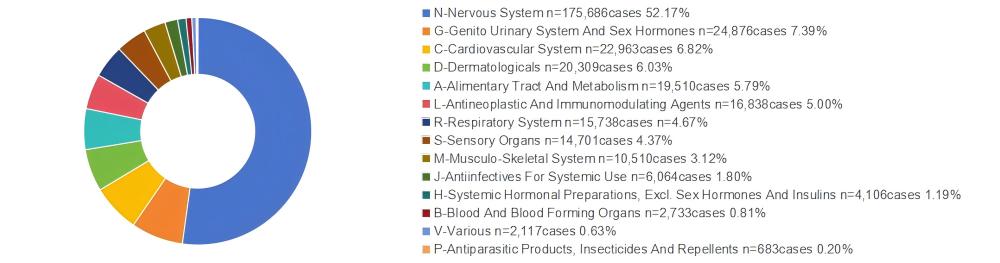


Supplementary Figure 1. Distribution of the number of cases of drugs with SAEs classified at ATC Level 1.


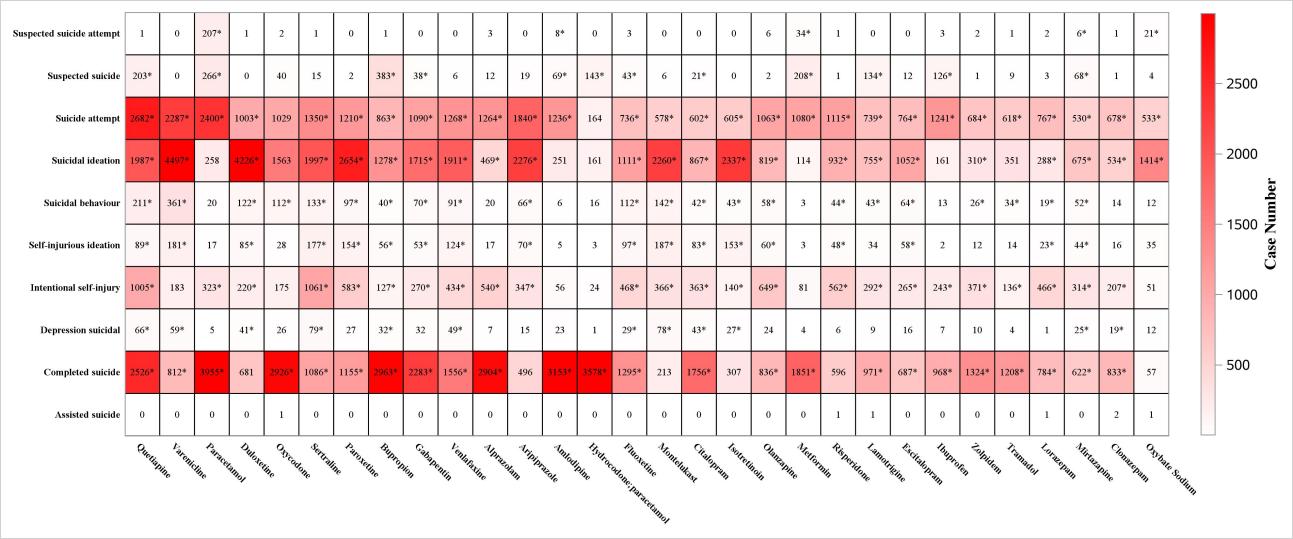


Supplementary Figure 2. Distribution of drugs and SAEs ranked in the top 30 by number of cases.

Note.* Indicates that the four methods are positive after detection.

# Supplementary Table

**Supplementary Table 1.**

| **Categories** | **Target adverse events reported** | **Other adverse events reported** | |
| --- | --- | --- | --- |
| **Target drugs** | a | b | |
| **Other drugs** | c | d | |
| **Methods** | **Calculation formula** | | **﻿Criteria** |
| **ROR** | ROR=ad/b/c | | a ≥ 3, 95%CI (lower limit) > 1 |
|  | 95%CI=e^ln(ROR)±1.96(1/a+1/b+1/c+1/d)^0.5^ | |  |
| **PRR** | PRR=a(c+d)/c/(a+b) | | a ≥ 3, 95%CI (lower limit) > 1 |
|  | *χ^2^*=[(ad-bc)^2^](a+b+c+d)/[(a+b)(c+d)(a+c)(b+d)] | | a ≥ 3, PRR ≥ 2, *χ^2^* ≥4 |
| **BCPNN** | IC=log_2_a(a+b+c+d)/(a+b)/(a+c) | | IC025>0 |
|  | 95%CI=E(IC)±2V(IC)^0.5^ | |  |
| **MGPS** | EBGM=a(a+b+c+d)/(a+c)/(a+b) | | EBGM05>2 |
|  | 95%CI=e^ln(EBGM)±1.96(1/a+1/b+1/c+1/d)^0.5^ | |  |

2× 2 tables and the calculation formulas and thresholds of the four methods.

Abbreviations: ROR, reporting odds ratio; PRR, proportional reporting ratio; BCPNN, Bayesian confidence propagation neutral network; MGPS, multi-item Gamma Poisson Shrinker; EBGM, Empirical Bayes Geometric Mean; 95% CI, 95% confidence interval; IC025, the lower limit of 95% CI of the IC; E(IC), the IC expectations; V(IC), the variance of IC; EBGM05, the lower limit of the 95%CI of EBGM.
